# Care–physical activity initiatives in the neighbourhood: study protocol for mixed-methods research on participation, effective elements, impact, and funding methods

**DOI:** 10.1186/s12889-018-5715-z

**Published:** 2018-06-28

**Authors:** Annemarie Wagemakers, Lisanne S. Mulderij, Kirsten T. Verkooijen, Stef Groenewoud, Maria A. Koelen

**Affiliations:** 1 Health and Society, Department of Social Sciences, Wageningen University, PO. Box 8130, 6700 EW Wageningen, The Netherlands; 2 Institute for Quality in Health Care (IQ Healthcare), RadboudUMC Nijmegen, P.O. Box 9101, 6500 HB Nijmegen, The Netherlands

**Keywords:** Health in all policies, Physical activity, Care, Effective elements, Socioeconomic status, Participation, Funding models

## Abstract

**Background:**

In the Netherlands, people with a low socioeconomic status (SES) live approximately 6 years less and are less engaged in physical activity (PA) than high SES citizens. This contributes to the persistent health inequalities between low and high SES citizens. Care–PA initiatives are deemed effective for stimulating PA and improving health and participation among peoples with a low SES. In those initiatives, multiple sectors (e.g. sports, health insurers, municipalities) collaborate to connect primary care and PA at neighbourhood level.

This study focuses on two Dutch municipalities that aim to invest in Health in All Policies (HiAP) and care–PA initiatives to improve the health of people with low SES. The aim is to gain insight into (1) the short-term (3 months) and long-term (1 year) outcomes of participating in care–PA initiatives for low SES citizens in terms of health, quality of life, and societal participation, (2) the effective elements that contribute to these outcomes, (3) the direct and perceived societal costs and benefits of care–PA initiatives, and (4) alternative ways to fund integrated care, prevention, and care–PA initiatives at neighbourhood level.

**Methods:**

The study will be built on a mixed-methods design guided by action research to continuously facilitate participatory processes and practical solutions. To assess outcomes, body measurements and questionnaires will be used as part of a pre-test/post-test design. Focus groups and interviews will be conducted to gain an in-depth understanding of outcomes and action elements. Action elements will be explored by using multiple tools: concept mapping, the logic model, and capacity mapping. Direct and perceived societal costs will be measured by administrative data from healthcare insurers (before-after design) and the effectiveness arena. An alternative funding model will be identified based on literature study, expert meetings, and municipal workshops.

**Discussion:**

Initiatives addressing multiple factors at different levels in an integral way are a challenge for evaluation. Multi-methods and tools are required, and data need to be interpreted comprehensively in order to contribute to a contextual insight into what works and why in relation to HiAP and care–PA initiatives.

## Background

Socioeconomic status (SES) is strongly related to health. In the Netherlands, people with high SES live approximately 6 years longer than people with lower SES [1]. Furthermore, people with high SES live approximately 19 years longer in good perceived health than people with lower SES [1]. Socioeconomic inequalities in health, or health inequities [2], are related to many causes of death and types of illness [3] and have proved to be persistent and seemingly unaffected by Dutch policy measures to date [1].

Although people in the Netherlands have become more physically active over the past years, those with low SES are less engaged in physical activity (PA) than high SES groups [4]. PA is an important contributor to health and well-being, and physical inactivity has been identified as the fourth leading risk factor for global mortality [5]. Health disorders associated with inactivity impose a substantial burden on societies and health systems [6]. In order to improve population health, to close the health gap between groups with higher and lower SES, and to reduce healthcare costs, the Dutch national government requires municipalities to implement Health in All Policies (HiAP) [7, 8], to provide care and PA close by, in the neighbourhood [7], and to stimulate citizens’ societal participation [9]. However, such policy and initiatives have not been evaluated comprehensively because of their complexity. Therefore, there is no insight into what works and why, i.e. what are the effective elements? Another question is how such initiatives should be funded. This study aims to get a comprehensive insight into HiAP, care–PA initiatives, societal participation, effective elements, and funding. Therefore, in the remaining part of this section, we address these topics and, subsequently, the research questions.

It is assumed that HiAP, in which sectors inside and outside the public health domain are made compatible, is effective in reducing socioeconomic health inequities [2, 10]. The approach explicitly emphasises that the promotion of health is the responsibility of all relevant sectors [11]. Therefore, different sectors are required to collaborate and reach a high level of agreement [12]; but this is challenging given, for example, differences in culture and interest [13]. It is recognised that multiple strategies across multiple levels are most effective in improving health and that there is a significant need for evaluation of such initiatives [14].

In care–PA initiatives, the primary care sector (e.g. physiotherapist, dietician, general practitioner) and the sport and the PA sector (e.g. sports club, fitness centres, PA lessons at community centres) collaborate with the aim of stimulating and maintaining PA among citizens who have, or are at risk of, chronic diseases such as diabetes and obesity. A recent literature review indicated that two different approaches in care–PA initiatives can be distinguished [15]. In the first approach, a primary care setting refers primary care patients to sport facilities through referral schemes. In the second approach, activities are organised by a network of primary care and sport professionals, for example a fitness centre that collaborates with primary care professionals to implement a programme. Care–PA initiatives focus primarily on prevention rather than on cure and are deemed effective for stimulating PA and improving health, quality of life, and (societal) participation among low SES citizens [12, 14].

In the Netherlands, participation in society (e.g. being employed, being part of a social network, or being a member of an association [16]) is emphasised by the Social Support Act [9], which came into force in 2007. Participation in society is considered crucial as it contributes to health by supporting the development of social capital and quality of life [17] and health and well-being [18]. Participation in health promotion initiatives contributes to the creation of supportive environments for health and the effectiveness of initiatives [19]. The World Health Organisation defines participation as one’s ‘involvement in a life situation’ ([20], p. 10).

The effective elements concept is often used interchangeably with similar concepts, e.g. principles for action as advocated and put centre core in current health promotion by the WHO and others [21, 22]. In this study, we use the effective elements concept as we aim to unravel the elements that make HiAP and care–PA initiative work to improve health and diminish health inequities. The underlying assumption of effective elements is that the effectivity of an initiative is caused by multiple principles or elements in combination. These elements are based on an ecological perspective on human health [23, 24], which emphasises the need for actions that are empowering [25], participatory [26, 27], intersectoral, equitable, and sustainable, and that use multiple strategies [28]. Moreover, effective elements relate to the capacity to develop and implement policy or initiatives that result in the desired output [29], emerge in practice, and depend largely on contextual factors and the knowledge and skills of the stakeholders involved [23].

The way care–PA initiatives need to be funded, especially for citizens with low SES, is a current topic of discussion, at both national and local policy level. Previous research has shown that willingness to pay (WTP), i.e. the maximum price one is willing to pay for example for health improvements [30], is limited [31]. A Dutch study among socially vulnerable groups found WTP for participating in a PA initiative to be 9.60 euro per month on average, and 16% were not willing to pay at all for sport and PA [31]. Therefore, it is important to address the question of who should pay for care–PA initiatives. Should this be participants or, for example, municipalities or health insurers? In the Netherlands, prevention is often not covered by health insurers, as current healthcare funding is based on fee for service systems (FFS) [32]. This means that healthcare providers are paid for the (curative) service they deliver, and this incentivises healthcare providers to increase their services (and, hence, healthcare costs) [32, 33]. In addition, citizens are not encouraged to take responsibility for their own health [34]. Alternative forms of funding (e.g. population-based funding) are promising as the focus is on citizens’ health, and possible savings are shared between healthcare providers [35]. In these alternative funding forms, stakeholders (healthcare providers, policymakers, insurers) need to collaborate, and perceptions need to be shifted to more positive conceptions of health, including patients’ societal participation [35]. Therefore, an important question to be addressed is what innovative funding methods are best to finance care–PA initiatives in order to enhance participation among socially vulnerable citizens and to contribute to limiting healthcare costs.

In this paper, we present a study protocol for a mixed-methods study to be implemented in two Dutch municipalities with the aim of gaining insight into strategies to develop, implement, and maintain HiAP and care–PA initiatives targeting citizens with low SES, the impact of these initiatives on outputs and outcomes, including societal participation, the effective elements that contribute to the output and outcomes, the perceived benefits of these initiatives, and alternative healthcare funding models. To our knowledge, all these components have not been studied in combination before. Therefore, four interrelated and successive research questions have been formulated:

1. What are the short- and long-term outcomes of low SES citizens’ participation in care–PA initiatives in terms of health, quality of life, and participation?
2. What are the effective elements contributing to the (expected) outcomes of care–PA initiatives?
3. What are the direct and perceived societal costs and benefits of care–PA initiatives?
4. What funding method is most adequate for strategies that provide integrated care, prevention, and PA at neighbourhood level?

## Methods/design

### Design

The study will be built on a mixed-methods design, i.e. a combination of quantitative and qualitative research methods, involving action research, a participatory process in which reflection results in action based on practical solutions [36]. Data will be collected in multiple rounds at the individual, group, professional, and municipal (including neighbourhoods and health insurers) level, through body measurements, questionnaires, focus groups, in-depth interviews, concept mapping [37], logic models [38], local public health capacity mapping [39], effectiveness arena [40], and the timeline method [41]. The body measurements and questionnaires will be administrated longitudinally, with a baseline measurement (T0) and two post-tests at 3 months (T1) and 1 year (T2). For the analysis of healthcare costs, a before-after design will be used, as participants’ data on healthcare consumption before the initiative started is available.

Multiple cases, i.e. five neighbourhoods in two municipalities, will be investigated within their real-life context. The individual case descriptions of the municipalities and neighbourhoods will enable a cross-case analysis to create more robust evidence than can be provided by a single case study [42]. The combination of information from multiple sources (e.g. policies, neighbourhoods, initiatives, different stakeholders’ perspectives) and multiple methods (e.g. body measurements, questionnaires, interviews, focus groups) increases the validity of the study by providing different options for triangulation of information [43].

Stakeholder involvement is key in this study, including citizens participating in care–PA initiatives, professionals from care, PA, and other relevant sectors (e.g. housing, welfare), and representatives from the municipality and neighbourhoods. In addition, regional and national organisations will participate in this study. For example, health insurers will participate as they support (financially) the care–PA initiatives in both municipalities, the Royal Dutch Society for Physical Therapy (KNGF) supports the collaboration of physiotherapists with other professionals in care–PA initiatives, and NLactief, the Dutch branch organisation for sport and PA centres, supports people with a chronic disease to become physically active in the neighbourhood.

### Conceptual model

**Fig. 1:**
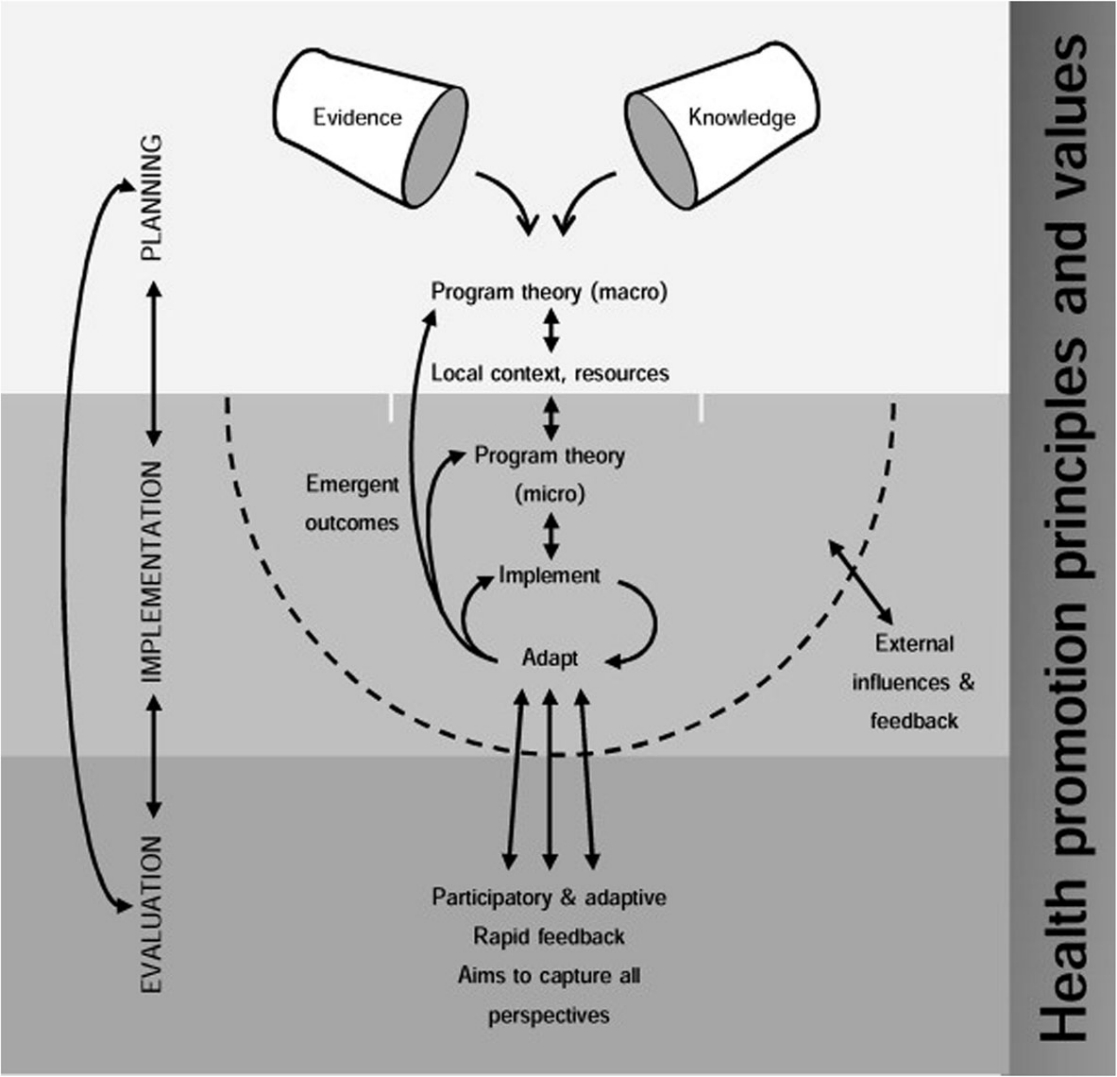
Jolley’s conceptual model of community-based health promotion [44]

In order to facilitate and evaluate care–PA initiatives comprehensively, Jolley’s conceptual model for community-based health promotion (CBHP) [44] will be used (Fig. 1). Jolley’s CBHP model can be seen as a helpful framework for designing the evaluation of complex CBHP programmes like care–PA initiatives [44]. An important principle in the model is the ecological perspective, which assumes that there are multiple levels of influence on health (intrapersonal, interpersonal, organisational, community, physical environment, and policy level) and that an individual’s health status and health-related behaviours are shaped by a dynamic interaction with the physical and social environment [24, 45]. This dynamic interaction between different levels makes the evaluation of interventions like current care–PA initiatives rather complex. To deal with this complexity, the CBHP model proposes that the different phases of a CBHP programme (i.e. planning, implementation, evaluation) be conducted in a non-linear (iterative) manner. The planning phase should yield a programme theory and logic model about how a programme is expected to work and what it will achieve. A programme theory encompasses the assumptions of involved actors, explaining how they expect the programme to achieve the desired outcomes [46, 47].

**Fig. 2:**
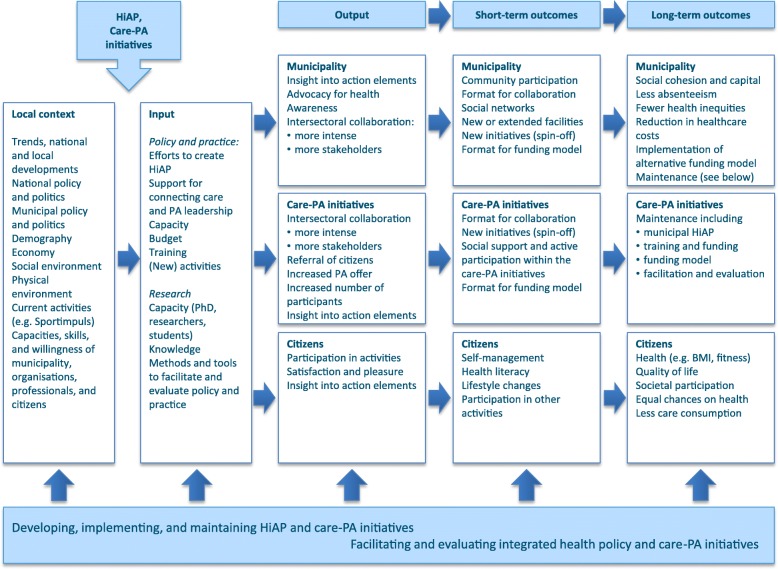
Logic Framework HiAP and care-sport initiatives

Based on programme theory, a logic model was constructed through group meetings in two municipalities with stakeholders and citizens prior to the start of this project (Fig. 2). The logic model functions both as a collective guide to plan and develop strategies and as a way to (scientifically) underpin and evaluate those strategies [38]. After the planning phase, the implementation phase should start with a more locally specific programme theory and logic model, taking context and resources into account. Next, the evaluation phase should aim to include the perspectives of different stakeholders, thereby being participatory. Rapid feedback from and to stakeholders should enable them to make changes to the programmes immediately (action research). Jolley stresses that, during all three phases, the local context of CBHP programmes (e.g. geographical area, economic/political factors, and so on) should be taken into account and that changes in the context should be recognised and acted upon.

### Study setting

The study will be conducted in and with Arnhem (155,699 inhabitants in 2017) and Veenendaal (64,273 inhabitants in 2017), two cities located in the centre of the Netherlands. This research focuses on deprived areas in Arnhem (Malburgen, Presikhaaf/Het Broek, Geitenkamp, Klarendaal) and Veenendaal (to be determined) that are characterised by an overrepresentation of socially vulnerable groups that are less physically active and score lower on quality of life compared to citizens in other neighbourhoods [48, 49].

Both cities are developing and implementing HiAP. In Arnhem, HiAP is based on a needs assessment among citizens in different neighbourhoods and aims to support care–PA initiatives targeting socially vulnerable groups [50]. Action plans to improve the quality of life in neighbourhoods have been developed, based on a so-called new integrated neighbourhood approach, thereby focusing on, among other things, joint (care) initiatives by citizens, social cohesion, citizen participation, and lifestyle coaches who guide citizens towards a healthier lifestyle, with a focus on PA [51]. Veenendaal aims to increase citizen participation, provide accessible and tailored facilities at neighbourhood level, and shift the focus from cure to prevention [52].

In both municipalities, collaboration between professionals, including primary care, is one of the main strategies. Also, both municipalities have several sport and PA facilities and initiatives, including a specialised PA centre in three deprived areas in Arnhem and one deprived area in Veenendaal, offering PA in combination with education on healthy lifestyles and social activities by a multidisciplinary team. One of these care–PA initiatives is X-Fittt (eXtra Frequency Intensity Training Time Transformation) 2.0.

### X-Fittt 2.0

X-Fittt 2.0 is a care–PA initiative for people with a minimum income that focuses on improving participants’ lifestyle and health. X-Fittt 2.0 is a combined lifestyle intervention, as multiple lifestyle behaviours, PA, and nutrition are addressed.

Inclusion criteria for participation in X-Fittt 2.0 are 1) having health insurance based on a minimum income via the municipality, 2) having a BMI ≥ 25 (kg/m^2^), 3) being ≥18 years of age, and 4) being motivated to partly pay for PA after the first phase of the programme.

X-Fittt 2.0 lasts 2 years (Table 1). In the first 12 weeks, participants are guided to live healthily by group sports sessions twice a week and an individual sports session once a week, dietary advice by a dietician, consultations with a physiotherapist, and lifestyle coaching by a lifestyle coach. After that, participants are encouraged to remain physically active by receiving lifestyle coaching for the remainder of the 2 years. Participants are regularly monitored on improvement in weight, BMI, waist circumference, fat percentage, and VO2max by a physiotherapist.

**Table 1:** Overview of X-Fittt 2.0 programme

	Phase 1: weeks 1–12	Phase 2: weeks 13–24	Phase 3: weeks 25–104
Participants	Start meeting with fittest (running, walking) (week 1) Participate in sports group twice a week Independent sports participation once a week	Continuation of PA, either at the PA centre or at another sports club/association of own choice	Continuation of PA, either at the PA centre or at another sports club/association of own choice
Lifestyle coach^a^	Intake: personal health check, lifestyle^b^, and development of plan with health and PA goals (week 0) Evaluation of progress throughout phase 1 Evaluation at the end of phase 1: discuss results and PA continuation	Evaluation of lifestyle, PA participation, and PA goals throughout phase 2	Evaluation of lifestyle, PA participation, and PA goals throughout phase 3 by phone
Physiotherapist^a^	Body measurements week 1 (T0)^c^ Body measurements week 12 (T1)^c^		Body measurements week 52 (T2)^c^
Sports coach	Provide training twice a week		
Dietician^a^	Dietary advice, one consultation	Dietary advice, one consultation	

^a^ The lifestyle coach has 4 h in phase 1, 2 h in phase 2, and 2 h in phase 3 for each participant. The physiotherapist has 2 h in phase 1 and 30 min in phase 3. The dietician has 1 h in phase 1 and 30 min in phase 2

^b^ Lifestyle data, which includes data on smoking, alcohol use, PA, employment and voluntary work, loneliness, and stress; data on individual participants’ PA goals will be obtained by the lifestyle coach at the intake of X-Fittt 2.0 and during multiple meetings with the participant over the 2 years

^c^ Body measurements will be taken as part of X-Fittt 2.0 and include height, weight, BMI, fat percentage, VO2max, blood pressure, waist circumference

X-Fittt 2.0 seems to be promising, based on a pilot study conducted in 2016 in Arnhem with 58 participants. Short-term outcomes indicate that, on average, participants lost 6.7 k of body weight during the first 3 months, and their self-reported health status improved from 6.0 to 7.3 (scale: 0–10). In addition, participants stated that their fitness improved and that their self-esteem increased [53]. Based on the successes so far, X-Fittt 2.0 will be continued in four neighbourhoods in Arnhem and in one neighbourhood in Veenendaal in 2018.

### Methods and tools

For each research question, the research activities and tools are explained in further detail in the following sections (see also Table 2). Research activities will be aligned with existing activities when possible. For example, focus group meetings will be organised in combination with meetings that already take place, and questionnaires will be administered simultaneously with other assessment occasions, i.e. the appointment with lifestyle coaches as part of X-Fittt 2.0 (Table 2). Furthermore, physiotherapeutic data of intake tests for the programmes will be used to research the impact of care–PA initiatives on the participants. Thus, the generated data will be mutually beneficial and pose a minimum burden for stakeholders and participants, thereby enhancing the efficiency and feasibility of this research. Self-report instruments will be assessed by Pharos (Dutch Centre of Expertise on Health Disparities) to align methods to the language used by X-Fittt 2.0 participants.

**Table 2:** Overview of research activities, tools, and output and outcome measurements at multiple levels

Level	Individual level	Group level	Professional and municipal level
Research question 1	Body measurements^a^ Height (cm) Weight (kg) BMI (kg/m^2^) Waist circumference (cm) Fat (%) VO2max Blood pressure (mmHG)	Focus groups (APEF tool) (T1 and T2) PA maintenance Motivation Societal participation Appreciation of X-Fittt 2.0 Appreciation of professional guidance Appreciation of PA in group	Concept model and logic model (see research question 2)
	Questionnaires^a^ Demographics (country of birth, education, household composition, daily activities, income) Lifestyle (smoking and alcohol) Quality of life (EQ-5D-3L, EQ-VAS) Diseases and healthcare use Monitoring of PA Societal participation (USER-P) Appreciation of professional guidance Appreciation of PA in group		
	In-depth interviews PA maintenance Motivation Societal participation Appreciation of X-Fittt 2.0 Appreciation of professional guidance Appreciation of PA in group		
Research question 2	In-depth interviews (see research question 1)	Focus groups (see research question 1)	Concept mapping and logic model Literature research 2 brainstorming sessions 5 interviews with HiAP experts Discussion in follow-up meetings
			Local capacity mapping Interviews with professionals Workshops in each municipality (Timeline technique)
Research question 3	X-Fittt 2.0 participants will be invited to effectiveness arenas		Direct costs analysis Description of costs and benefits Estimation average costs per activity Healthcare consumption
			Perceived benefits and costs 5 focus groups (effective arena)
Research question 4			Alternative funding model Literature research to identify models 2 expert meetings to choose model 2 workshops (in each municipality)

^a^ Data collection for body measurement and questionnaires will be conducted for each group at T0 (start of X-Fittt 2.0), T1 (12 weeks after the start of X-Fittt 2.0), and T2 (1 year after the start of X-Fittt 2.0). Body measurements will be taken by the physiotherapist as part of the X-Fittt 2.0 programme. All other data will be collected by the researchers

*Abbreviations: EQ-5D-3L* EuroQol 5-dimensions 3-levels, *EQ-VAS* EuroQol visual analogue scale, *APEF* activate participation, enjoyment, and fostering group processes, *USER-P* Utrecht scale for evaluation of rehabilitation-participation

### Research question 1: Outcomes in terms of health, quality of life, and societal participation

To assess the outcomes of low SES individuals’ participation in X-Fittt 2.0, body measurements, information on lifestyle and PA, and questionnaires will be used as part of a pre-test/post-test design. This will be administered at the start of the programme (T0), after 3 months (T1), and after 1 year (T2). Furthermore, focus groups and interviews will be conducted to gain in-depth insight into the short-term and long-term outcomes on health and societal participation.

#### Body measurements, lifestyle, and PA

Body measurements include height, weight, BMI, fat percentage, VO2max, blood pressure, and waist circumference and will be measured by a physiotherapist as part of X-Fittt 2.0. Height is measured to the nearest 0.1 cm with a measuring tape, and weight is measured to the nearest 0.1 kg. Participants are measured with light clothing and no shoes. BMI scores are calculated based on height and weight. Waist circumference is measured with a measuring tape to the nearest 0.1 cm. Fat percentage is measured by measuring skin fold thickness (biceps, triceps, subscapular, suprailiac) using the Slim Guide Skinfold Caliper C-120 [54]. VO2max is measured with the Åstrand/Ryhming cycle test and a heartrate monitor chest strap [55], and blood pressure is measured with a sphygmomanometer.

#### Questionnaires

The standardised questionnaire topics to measure short- and long-term outcomes are demographics, lifestyle, quality of life, diseases and healthcare use, monitoring of PA, motivation, societal participation, appreciation of the professionals, and appreciation of PA in a group.

Demographic information about participants will be obtained by questions on age, sex, country of birth, highest level of education completed, present household composition, main daily activities (e.g. work, volunteering, housekeeping), and income. Data on sex, country of birth, highest level of education, and income will be collected only at T0.

Lifestyle is assessed with four questions: two about smoking behaviour (yes/no and number of cigarettes each day) and two about alcohol use (yes/no and number of glasses each day/week/month).

To measure health-related quality of life, the Dutch EuroQoL 5 Dimensions 3 Level scale (EQ-5D-3L) and the EQ visual analogue scale (EQ-VAS) will be used. The EQ-5D-3L is a standardised measure of health status that provides a simple, generic measure of health [56]. The EQ-5D asks respondents to describe their health in terms of the level of problems (no, some, or extreme) on each of the five dimensions: mobility, self-care, usual activities, pain/discomfort, and anxiety/depression. To make the questions more suitable for our study population, the formulation of the questions and answer options have been adjusted to meet the level of the participants in collaboration with, and as suggested by, Pharos. The EQ-VAS is a vertical visual analogue scale that takes values between 100 (the best imaginable health) and 0 (worst imaginable health) on which respondents provide a quantitative assessment of their health [56]. The scale was changed to a horizontal scale, as suggested by Pharos.

Disease and healthcare use will be measured by questions about diseases in a certain period (depending on whether the questionnaire is filled out in T0, T1, or T2), medicine intake, contact with general practitioner, and contact with other care providers that are not connected to X-Fittt 2.0.

Participants will be asked to indicate whether or not they monitor their own PA behaviour; and, if they do so, they have to indicate how they monitor this.

To measure and to unravel the influence of care–PA initiatives on societal participation, first the concept of participation has been further operationalised based on the participation wheel [57] and scientific literature [5, 19, 58–60]. Social levels of participation include for example ‘interacting with others, doing an activity with others, helping others, and contributing to society’ ([60], p. 2148). The participation wheel, developed in the Netherlands to guide promotion of participation and associated legal frameworks, also shows several dimensions of societal participation, ranging from employment, volunteering, and caring for others to meeting with others and being able to self-manage life [57]. Second, based on this conceptualisation of participation, the Utrecht Scale for Evaluation of Rehabilitation-Participation (USER-P) [61] has been selected as a measurement instrument, as this fits best the operationalised dimensions. The USER-P is a generic and valid instrument to rate objective and subjective participation in persons with physical disabilities with a good responsibility compared to other participation measures [62, 63]. The original questionnaire consists of three parts: (1) time spent on, and frequency of, daily activities, like working, studying, household, and going out, (2) restrictions in daily activities, and (3) satisfaction with daily activities [61]. For the purposes of this study, only part 1 and part 3 are included in the questionnaires. In part 1 of the original set of questions, six answer options are provided to indicate the frequency of the different daily activities in the previous 4 weeks. On Pharos’s recommendation, this has been decreased to four answer options (every day, a few times a week, once a week, never) to indicate the frequency of the different daily activities over a regular week in our questionnaire, to fit the participants’ level. Part 3 originally consisted of six answer categories to indicate satisfaction with different daily activities. This has been narrowed down to four answer categories (I am happy, I do not care, I am unhappy, not applicable) in our questionnaire.

Questions about appreciation of the lifestyle coach, physiotherapist, dietician, and physical exercise trainer will be asked to measure the appreciation of professional guidance in the programme (3-point scale: good, normal, and bad). For each professional, there is space for adding the reason for the level of appreciation. These questions will be asked only at T1 (for all professionals) and T2 (only for the lifestyle coach), as the participants do not yet have experience with the programme at T0.

Finally, appreciation of PA in a group will be measured by five items on a 3-point scale, covering enjoyment, motivation, appreciation, and influence of the group, and exchanging experiences. This will be measured only at T1, as PA in the X-Fittt 2.0 group stops after T1.

#### Sample size and power

The impact of X-Fittt 2.0 on physiological and self-report measures will be assessed by means of a one-group pre-test/post-test design. Because participants cluster within different X-Fittt 2.0 groups that cluster within different municipalities, multilevel analysis will be used to analyse the data. Sample size calculation for multilevel modelling is complex however, and estimates derived from available software tend to have limitations [64]. Because the primary aim of our research is to measure effects at the participant level, which makes the number of participants key to obtain sufficient statistical power, it was decided to conduct a power analysis based on a relatively simple paired sample t-test. The power calculation was based on the weight variable, as weight loss is a primary outcome of X-Fittt 2.0 and inclusion is based on BMI. Estimation of effect size was based on pilot data from X-Fittt 2.0 (*n* = 36), which revealed that, on average, participants lost 6.7 k of body weight (SD = 4.9) during the first 3 months of the programme [53]. The sample size calculation was conducted with G*Power version 3.0.10 with alpha set on 0.05, a power of 0.80, and a rather conservative effect size of 5 kg with a standard deviation of 5. This led to a required sample size of 8. Given the drop-out rate of 26% in the pilot programme X-Fittt 2.0 [53] and a drop-out rate of 40% in a Dutch community-based PA programme also targeting socially vulnerable groups with four measurements (drop-out rate 40%) [65], a drop-out rate of 40% is assumed. The required number of participants to obtain reliable estimates of mean weight loss is therefore 14. On average, X-Fittt 2.0 groups consist of 10 participants. The aim is to include at least 15 X-Fittt 2.0 groups across the five neighbourhoods, resulting in a total final sample of at least 90 participants.

#### Focus groups and in-depth interviews

The short-term and long-term impact of X-Fittt 2.0 will also be assessed by means of focus groups (T1, T2) and in-depth interviews (T2) with X-Fittt 2.0 participants. Topics to be addressed in the focus groups and in-depths interviews include PA maintenance, motivation, societal participation, effective elements (to be identified in research question 2), and appreciation of the X-Fittt 2.0 programme, professionals’ guidance, and doing PA in a group.

Statements in focus groups and items in interviews on societal participation will be based on the operationalisation of societal participation as explained before. Statements and items about motivation will be based on the Integrated Change (I-Change) model, derived from the attitude–social influence–self-efficacy model, which can be considered as an integration of various theories [66]. The I-Change model states that behaviours are determined by a person’s motivation or intention to carry out a particular type of behaviour. Three main types of factors determine a person’s motivation: attitudes, social influences, and self-efficacy expectations.

For the focus groups, the Activate Participation, Enjoyment, and Fostering (APEF) group processes tool [23, 67] will be used. Existing statements in the tool will be adapted or replaced to fit operationalisations of PA maintenance, societal participation, main types of factors of the I-Change model, and appreciation of X-Fittt 2.0, professional guidance, and PA in a group. The APEF tool was originally developed to assess participants’ perceptions on group-based principles for action and consists of statements on which participants in groups vote, followed by an in-depth discussion. The voting procedure engages participants, and spider diagrams visualise participants’ perception of the statements. The APEF tool addresses the challenge of relating group level outcomes to individual outcomes such as PA behaviour and motivation. The tool facilitates as well as evaluates group-based principles for action, it stimulates dialogue and is culturally sensitive, but it needs strong facilitating skills to manage group dynamics [67].

Focus groups will be held with all X-Fittt 2.0 groups participating in the research. Inclusion of all X-Fittt 2.0 groups in focus groups stimulates participation and might contribute to participants’ motivation to continue PA in groups.

Topics in the in-depth interviews will be addressed by open questions in order to explore participants’ perceptions and experiences. Interviews will be conducted with four to six participants from each group to get a broad and complete insight into perceptions and experiences while also being able to get insight into differences between groups, neighbourhoods, and municipalities.

Focus groups and interviews also contribute to the identification of effective elements (research question 2).

### Research question 2: Identification of effective elements

The effective elements concept refers to the assumption that the effectivity of an initiative is caused by multiple ele-ments. Effective elements can be further distinguished into elements that comprise the core of the initiative, core effective elements, and elements that are more context-specific, specific effective elements [68, 69]. In this study, both core and specific effective elements will be unravelled. Concept mapping, logic models, and capacity mapping are promising tools to deal with complexity and to gain insight into effective elements at the municipal level. Effective elements within groups will be explored by analysis of the focus groups and interviews with X-Fittt 2.0 participants (see also research question 1).

#### Concept mapping and logic model to conceptualise effective elements

Concept mapping will be used to conceptualise and visualise effective elements by generating, structuring, interpreting, and utilising statements in the form of a concept map [37]. Concept mapping is a standardised tool for developing a conceptual framework of a complex topic and has already been used for a wide variety of subjects, including health promotion [37, 70]. The logic model will be used to operationalise and map the effective elements in relation to input, output, and outcomes [38].

Effective elements will be operationalised and identified in four steps. First, literature research (journal articles, grey literature) on (indicators of) effective elements and input, output, and outcome indicators will be identified and formulated into statements and included in a provisional logic model for each municipality, based on the overall logic model for the project (Fig. 1). Second, in each municipality, statements and the provisional logic model will be discussed and adapted through brainstorming sessions with local stakeholders at regular meetings (existing or organised by the project) and, third, through interviews with five national experts in HiAP and/or care–PA initiatives. Finally, in a follow-up meeting, results will be shared with stakeholders, and subsequent actions for policy and practice will be discussed in each municipality.

#### Local public health capacity mapping

Public health capacity encompasses the organisational, human, financial, and other resources that enable action to be taken by responsible authorities to improve health and reduce health inequalities [71]. Capacity mapping is a tool that can be used to identify these resources. However, there is as yet no consensus on the main dimensions of public health capacity [72]. In previous research, a capacity mapping framework for the work of Care Sport Connectors was developed [39] based on Aluttis et al.’s country level framework for public health capacity [72], Meyer et al.’s conceptual model for public health systems and services research [71], and Bagley and Lin’s rapid assessment tool for public health system capacity [73]. In this project, the framework will be further adapted to the local context, and the focus will be broadened to include not only public health capacity but also capacity delivered by other sectors. To map local capacity for public health, prevention, and care–PA initiatives, and to observe potential change over time, interviews with professionals in the care–PA initiatives and municipal sectors will be conducted in 2018 and 2020. In addition, group level techniques for assessment will be used in order to document the collectively experienced benefits. In 2018 and 2020, workshops, as part of regular meetings with municipal stakeholders, will be organised to discuss local capacity for public health, prevention, and PA promotion. In 2020 also, a timeline technique [41] will be used as a reflective tool to provide a comprehensive, historical, and context-specific understanding of developments in policy and care–PA initiatives in both municipalities.

### Research question 3: The direct and perceived societal costs and benefits of care–PA initiatives

The rationale for studying the actual and perceived societal costs is to find and document justification for a certain project. Justification is derived when all expected benefits, costs, and alternative options have been carefully considered and prove supportive of the proposed project, i.e. X-Fittt 2.0. The focus in this study will be on direct and perceived costs and benefits. Indirect costs, for example costs that have been incurred for infrastructure and collaboration by different sectors, which function as a prerequisite for care–PA initiatives, are sunk costs that cannot be retrieved. Direct costs are costs incurred to implement the programme, for example treatment of patients by primary care professionals or referral to, and treatment by, secondary care, but also intake at a sports facility.

Direct costs, in terms of benefits and cost savings, will be calculated largely by using existing data (e.g. data from Statistic Netherlands (CBS) and claims data from healthcare insurers). This cost analysis will be based on two elements: 1) a description of the average HiAP and care–PA pathway, i.e. the bundle of activities undertaken for HiAP and care–PA initiatives and 2) estimation of average costs per activity, based on the Dutch guidelines for economic evaluations in healthcare [74]. Measuring benefits in terms of cost savings is based on the assumption that HiAP and care–PA initiatives will cause less healthcare consumption in both the primary and the secondary care sector in the long run.

Administrative claims data from healthcare insurers at two points in time (before-after design) of X-Fittt 2.0 participants will be used in order to compare healthcare consumption before and after participation in X-Fittt 2.0. To maintain anonymity and to take into account the privacy regulations, data on healthcare costs will be sent to a trusted third party (TTP). This TTP will combine the health insurers’ data with data collected by the researchers of this project and provide us with an anonymised dataset that we can use to answer the third research question. All participants will be asked for written consent to use their claims data.

Perceived costs and benefits will be assessed by an effectiveness arena as this can add a richer and fuller understanding to the hard figures of costs and benefits of care–PA initiatives. The Dutch *EffectenArena* [75] is a tool designed and validated in practice to obtain, with stakeholders, a better insight into the value of societal programmes. The tool has proved useful in joint decision-making processes because it helps to make explicit the expectations that individual partners hold towards the effects of a programme and the specific actions that lead to these effects. By sharing and discussing these thoughts, stakeholders gain new insights. In 2020, in each of the five neighbourhoods in the two municipalities, stakeholders and citizens (X-Fittt 2.0 participants) will be invited to a focus group discussion in which they will be challenged to make explicit connections between the actions undertaken as part of HiAP and care–PA initiatives and the societal effects that they have in mind.

### Research question 4: The most adequate funding method for integrated care, prevention, and PA at neighbourhood level

Recently, there has been much debate on the best ways to fund healthcare. Originally, FFS dominated the spectrum. However, several disadvantages have been reported [34]. One important disadvantage is that FFSs incorporate the incentive for healthcare professionals to do more: more healthcare generates more income for them. Hence, citizens are not encouraged to take care of themselves, live healthily, and try to avoid healthcare consumption. Therefore, alternative forms of funding have been proposed. In the US for example, experiments have been conducted with accountable care organisations that were funded depending on the health of ‘their citizens.’ In Germany, the Gesundes Kinzigtal experiment did the same; and, in the UK, healthcare commissioning groups are going in the same direction. Population-based funding has one essential feature: possible savings – because people become healthier and healthy people use less healthcare – must be shared between purchasers/payers and healthcare providers: so-called shared savings constructions. Otherwise, the incentive that should encourage providers to innovate will not function.

In this study, following on from the literature, we will elaborate further on existing and new funding models. For example, the OECD proposes three innovative funding methods that can lead to more efficiency in healthcare, cost reduction, and improved quality of healthcare [32]: i) population-based payments, in which a group of healthcare providers provides high-quality healthcare, while keeping the costs below a certain benchmark; ii) add-on payments, in which payments complement existing funding methods, for example ‘pay-for-performance’, which are add-on payments promoting prevention and meeting certain performance measures; and iii) bundled payments in which multiple services for a certain condition (e.g. diabetes) are grouped together for payment [32]. Next, in two expert meetings, we will rank alternative funding models based on criteria in discussion with the stakeholders, in particular healthcare insurers. Finally, we will select one preferred alternative funding model and discuss this model in a workshop in each municipality with local and national stakeholders and list the (evidence-based) benefits and challenges of the chosen alternative funding methods, resulting in recommendations for implementation.

### Data analysis

Quantitative data derived from the body measurements and questionnaires will be analysed using R packages on the basis of descriptives (e.g. means and frequencies), t-tests, or – in the event of skewed data distributions – non-parametric alternatives, and by multilevel techniques.

Qualitative data, focus groups, (in-depth) interviews, brainstorming sessions, discussion meetings, workshops, expert interviews, and meetings will be recorded and transcribed verbatim. Data will be analysed using Atlas ti.8 software. The transcriptions of all qualitative data will be coded by two researchers. Discrepancies will be discussed until agreement is reached. Different analysis techniques will be applied, depending of the nature of the data. For example, the in-depth interviews to explore participants’ perceptions will be analysed inductively.

In order to gain a comprehensive and contextual insight into what works and why, realist synthesis [46] will be used to identify key combinations of contextual factors and mechanisms that trigger outcomes of interest. A realist synthesis starts with an account of processes that explains how a programme leads to a particular outcome. The focus is on context–mechanism–outcome (CMO) configurations. For instance, the analysis of qualitative data from interview transcripts may be based on coding in terms of ‘outcomes as observed by respondents,’ ‘context conditions’, and ‘underlying mechanisms – or effective elements – in the actual programme.’ The final research output from realist synthesis is not a statement of effect size, as the same programme will have different effects in different contexts, but a refinement of the programme theory. Previous use of realist synthesis in the project Communities on the Move provided a rich and detailed understanding of mechanisms at programme level [76].

## Discussion

Health inequalities between low and high SES citizens continue to exist in the Netherlands. As low SES citizens constitute a vulnerable group in society, evidence-based strategies are needed to improve their health and to reduce health inequalities. This fits with the goals of Dutch national health policy, which aims to increase citizen participation [11] and connect and provide care and PA in the neighbourhood [10]. The aim of the study described in this protocol is to gain a comprehensive and contextual insight into what works why in relation to HiAP and care–PA initiatives that aim to promote physical activity among citizens with low SES and to reduce health inequalities. The project will be conducted in Arnhem and Veenendaal, two municipalities in the Netherlands that aim to improve the health of low SES citizens and support care–PA initiatives targeting socially vulnerable groups from a bottom-up point of view.

HiAP and care–PA initiatives have been implemented relatively recently, and consequently little research has been conducted to evaluate them comprehensively. Therefore, a multi-case and multi-methods design is proposed. We will follow deprived areas over time in their real-life context, and therefore the research can be viewed as a natural experiment. Monitoring real-life interventions, however, also imposes challenges for evaluation, as traditional research criteria, such as objectivity of the inquirer, systematic rigour of fieldwork procedures, and generalisability of findings, are not easy to apply. Furthermore, drop-outs from a care–PA initiative are hard to follow up, as found during the pilot study.

What adds to the complexity is that we aim to analyse relevant processes and outcomes, at multiple levels, not in isolation, but in connection with one another. This is challenging. However, certain strategies are foreseen that will assist the data analysis. First of all, the logic framework that will be (further) developed for this research will help to identify and define processes and output and outcome indicators at different levels, and hence help to gain and retain a clear view of the project. Second, action research will be a prominent strategy and will be used to engage different stakeholders, including socially vulnerable groups, in order to stimulate change to improve practice and to contribute to generating an evidence base of what works why in a real-life setting [19]. Engaging stakeholders improves the external validity of the research, that is, its applicability and usability in other settings [19, 36]. Furthermore, the value of action research is that it reflects the values of health promotion, such as participation and empowerment [77], enables those involved to continually optimise their strategies, and contributes to (further) developing theories and (other) research methods [19]. Finally, constructivist evaluation criteria will be used in developing our methods for quantitative as well as qualitative data collection, such as acknowledging subjectivity, capturing and respecting multiple perspectives, doing justice to the integrity of unique cases, contributing to deepening understanding of dialogues, and engaging those with less power respectfully and collaboratively.

## Abbreviations

APEF: Activate Participation, Enjoyment, and Fostering group processes
CBHP: Community-based health promotion
CMO: Context–mechanism–outcome
EQ-5D-3L: EuroQol 5-Dimensions 3-Levels
EQ-VAS: EuroQol visual analogue scale
FFF fee: For service systems (FFS)
HiAP: Health in All Policies
KNGF: Royal Dutch Society for Physical Therapy
PA: Physical activity
Pharos: Dutch Centre of Expertise on Health Disparities
SES: Socioeconomic status
TTP: Trusted third party
USER-P: Utrecht scale for evaluation of rehabilitation-participation
WTP: Willingness to pay
X-Fittt: eXtra frequency intensity training time transformation
